# Dressmakers show enhanced stereoscopic vision

**DOI:** 10.1038/s41598-017-03425-1

**Published:** 2017-06-13

**Authors:** Adrien Chopin, Dennis M. Levi, Daphné Bavelier

**Affiliations:** 1 Ecole Normale Supérieure, Paris Sciences et Lettres Research University, CNRS, Département d’études cognitives, Laboratoire des Systèmes Perceptifs, Paris, France; 2 Faculty of Psychology and Educational Science, University of Geneva, Geneva, Switzerland; 3 School of Optometry, and Helen Wills Neuroscience Institute, University of California, Berkeley, Berkeley, CA USA; 4 Brain and Cognitive Science Department and Center for Visual Science, University of Rochester, Rochester, NY USA

## Abstract

The ability to estimate the distance of objects from one’s self and from each other is fundamental to a variety of behaviours from grasping objects to navigating. The main cue to distance, stereopsis, relies on the slight offsets between the images derived from our left and right eyes, also termed disparities. Here we ask whether the precision of stereopsis varies with professional experience with precise manual tasks. We measured stereo-acuities of dressmakers and non-dressmakers for both absolute and relative disparities. We used a stereoscope and a computerized test removing monocular cues. We also measured vergence noise and bias using the Nonius line technique. We demonstrate that dressmakers’ stereoscopic acuities are better than those of non-dressmakers, for both absolute and relative disparities. In contrast, vergence noise and bias were comparable in the two groups. Two non-exclusive mechanisms may be at the source of the group difference we document: (i) self-selection or the fact that stereo-vision is functionally important to become a dressmaker, and (ii) plasticity, or the fact that training on demanding stereovision tasks improves stereo-acuity.

## Introduction

Depth perception is an important human visual ability allowing people to interact easily with their environment. It relies substantially on the stereoscopic depth information, which itself is based on image binocular disparities. These disparities are caused by the different viewpoints of the two eyes. Monocular cues to depth (e.g. motion parallax, shadows, occlusion) also contribute to depth perception^1^.

The functional role of stereopsis has been the subject of much debate. It has been theorized to guide the fine movements of the hands in reaching and grasping^2–4^. Indeed, object placement^5, 6^ and grasping^7–10^ are more precise with binocular viewing than monocular viewing (at least in the centre of the visual field^11^). However, most of the evidence is based on comparing binocular and monocular viewing conditions, which differ not only in the absence of stereopsis, but also in an absence of binocular vergence and summation, and a decreased field of view. It is known that decreasing the field of view affects reaching^12^. Yet, there remains a binocular advantage in object prehension even when controlling for the field of view^13^. There is also a growing body of confirmatory evidence, including studies showing that binocular cues to depth are crucial to prehension^14^, that binocular cues are given more weight than monocular cues when placing objects^15^, and that the binocular advantage in object placement correlates with stereo-acuity^5^. Previous studies^6, 16^ have shown that binocular vision is more efficient than monocular vision in delicate manual tasks like threading a needle. Some have argued that stereopsis can only be useful for slow motions requiring extreme precision^17^. However, past studies have not shown better stereo-acuities for professions based on slow motions requiring extreme precision, like surgeons^18^ or dentists^19, 20^, although stereoblind surgeons performed a simulated surgical task significantly worse than the stereo-normal ones^21^. Furthermore, stereo-acuity when entering a school of dentistry was not linked with later student grades^22^.

In the current study, we tested stereoscopic acuities of a sample of dressmakers, and compared these acuities with those of a non-dressmaker group. Given the likely advantage given by stereopsis in fine eye-hand tasks, we reasoned that dressmakers may display better stereo-acuities. This could result either through self-selection or through the development of expertise given that their daily work involves constantly estimating small changes in visual depth. Indeed, stereoscopic vision is known to undergo some training-dependent plasticity. For example, stereo-perception can be ameliorated by training on a depth task with random dot stereograms^23–25^, or a depth task with local stereograms, involving edges, squares, lines, dots, or Gabor patches^24, 26–29^. In addition, persons with strabismus and amblyopia, who often suffer from stereo-blindness, have been trained to recover stereoscopic vision with various rates of success (for a review, see ref. 4), using techniques such as patching^30^, monocular^31^ or dichoptic perceptual learning^32, 33^, monocular^34^ or dichoptic video gaming^30, 35, 36^, and stereo-training^37–39^. However, it is not known whether manual actions, in particular, the kind of fine actions involved in sewing can increase stereoscopic depth perception, or whether having poor (or no) stereopsis would deter individuals from professions such as dressmaking.

Although we have discussed stereoscopic acuity as if it were a unitary concept, it is well known that there are two different types of disparity: absolute disparity and relative disparity.

**Figure 1:**
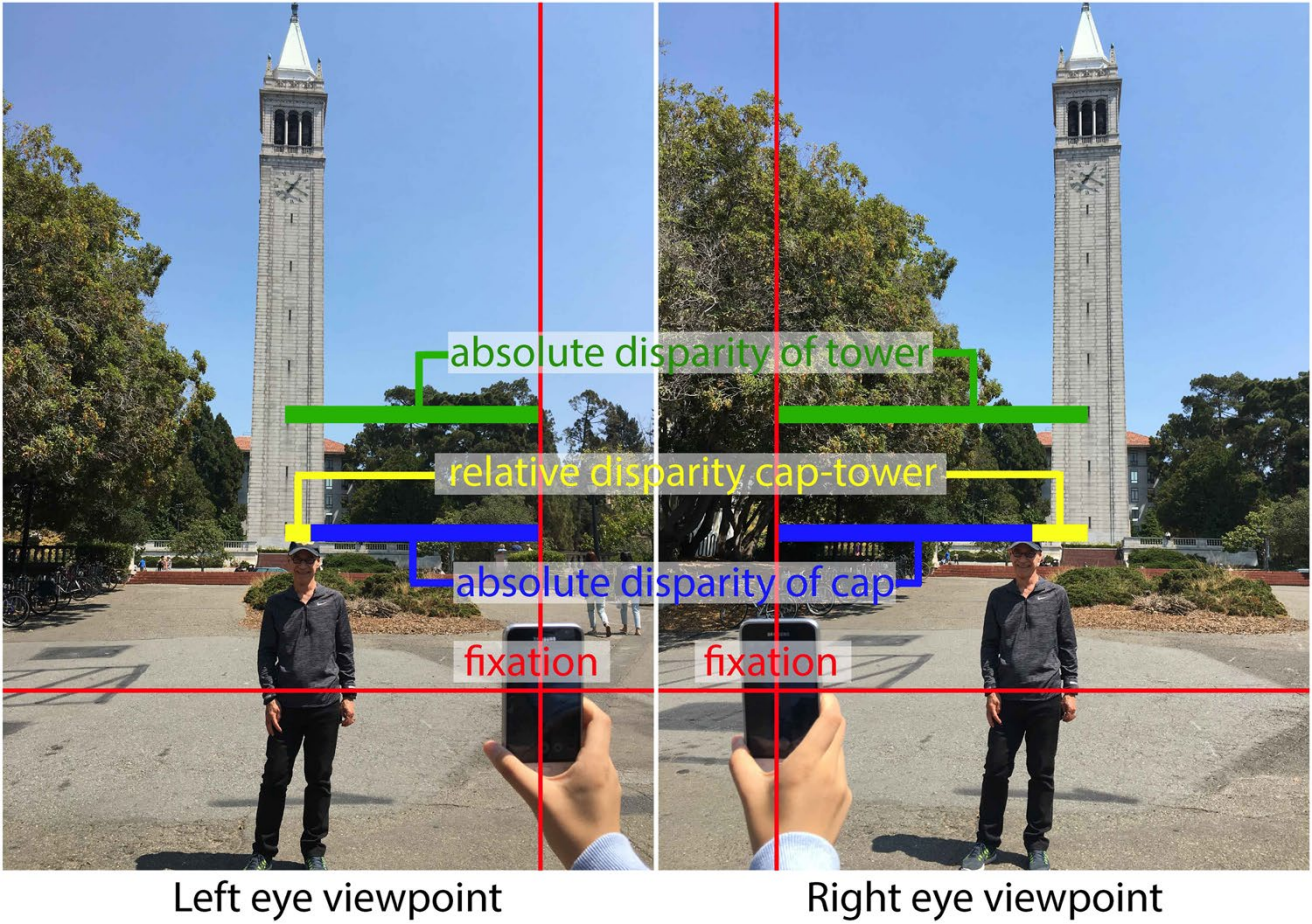
Schematic illustration of absolute and relative disparities. Left and right panels show the viewpoints from left and right eyes respectively. The observer fixates on the phone (fixation indicated in red crosshairs). The absolute disparity of the author’s cap is the sum of the distances indicated in blue while the absolute disparity of the tower (Berkeley’s campanile) is the sum of the distances indicated in green. The relative disparity between the cap and the tower is the sum of the distances indicated in yellow, and also the differences of the absolute disparities of the cap and of the tower. A more formal definition can be found in ref. 41.

An object’s absolute disparity is the difference between the angle subtended by the target at the two entrance pupils of the eyes and the angle of convergence. Absolute disparity is important for judging the depth distance of an object from one’s self (Fig. 1). The difference between the absolute disparities of two objects is called relative disparity (Fig. 1). Relative disparity is important for judging the depth distance between two (or more) objects. It is well known that human observers are better at judging relative disparity than at judging absolute disparity^40^. We and others have argued that the source of this difference is an absence of conscious readout for absolute disparities. We refer to this as the absolute disparity anomaly^41^. Despite this anomaly, humans should have a high sensitivity for absolute disparities, given that both vergence eye movements^42–45^ and relative disparities are based on absolute disparities^41, 46, 47^. The plasticity studies discussed above were all conducted with relative disparities. Therefore, it is not clear whether absolute disparity acuity (or readout) can be improved by learning. On the one hand, in a recent study^41^, we have found very little evidence for rapid learning of absolute disparity sensitivity (or readout), suggesting it may be difficult to change. However, our participant sample was small (n = 6) and we tested learning over only 1200 trials. On the other hand, given the assumed link between absolute disparities and relative disparities, at least under the absolute disparity anomaly view, changes in relative disparity could go hand in hand with changes in absolute disparities. Therefore, we tested both absolute and relative disparities, in order to learn whether expertise in sewing might be associated with better relative or absolute disparity acuity (or readout), or both.

Absolute disparity is a cue for vergence. However, it is widely believed that relative disparity acuity is considerably better than absolute disparity acuity, because absolute disparities are corrupted by vergence noise^2, 42, 48^. In a recent article^41^, we argued against that idea by showing that vergence noise was too small to explain the difference between absolute and relative disparity acuities. Rather, we suggested that vergence noise is not the limiting factor for absolute disparity measurements. Given that debate, however, we felt it was important to measure vergence ability. Furthermore, we were interested to learn whether dressmakers (who need to converge accurately) would show less vergence noise than non-dressmakers. For that purpose, we measured vergence noise and bias (over-convergence or divergence during fixation) for each participant with the Nonius-line technique.

## Results

We compared absolute and relative disparity thresholds of dressmakers and non-dressmakers. In addition, we measured vergence thresholds under nearly identical conditions and separated the results in two values: fixation noise and fixation bias. All stimuli were briefly presented to minimize eye movements.

### Stereo-thresholds: Dressmakers are better than non-dressmakers

A mixed-model ANOVA on Log-thresholds with group as between-subject factor and disparity task (absolute/relative) as a within-subject factor established a main effect of task (F(1,32) = 67; p < 10^−5^), and importantly of group, with the dressmakers outperforming the non-dressmakers (F(1,32) = 6.2; p = 0.018; the interaction “disparity task × group” was not significant, p = 0.99).

**Figure 2:**
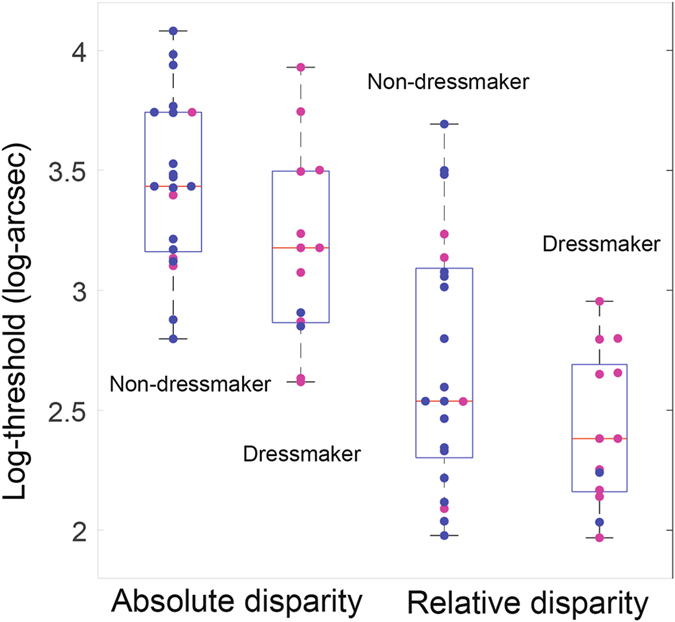
Boxplots of log-transformed thresholds for discrimination of depth from absolute disparities only (left side) and from additional relative disparities (right side), for non-dressmaker and dressmaker groups. The median for each group is in red and the blue box defines the Q1 and Q3 quantiles for each group. The whiskers encompass the entire distribution. Each pink dot is a data point for a female participant and each blue dot is a data point for a male participant.

As illustrated in Fig. 2, the dressmakers displayed better (i.e., lower) absolute (1504 vs. 2714 arcsec; T(32) = 1.78; p = 0.05) and better (i.e., lower) relative disparity acuities (241 vs. 345 arcsec; T(32) = 2.16; p = 0.025) than the non-dressmakers (one-sided post-hoc t-tests with Holm-Bonferroni-corrected p-values for the between-group differences). The effect sizes were relatively small (for the absolute disparity task: Cohen’s d = 0.68; for the relative disparity condition: Cohen’s d = 0.34), mostly because of the large range and variance of performances: dressmakers’ median acuity was 43% better in the relative disparity condition and 80% better in the absolute disparity condition, when compared to non-dressmakers’ acuity.

### Vergence noise and bias do not differ between dressmakers and non-dressmakers

**Figure 3:**
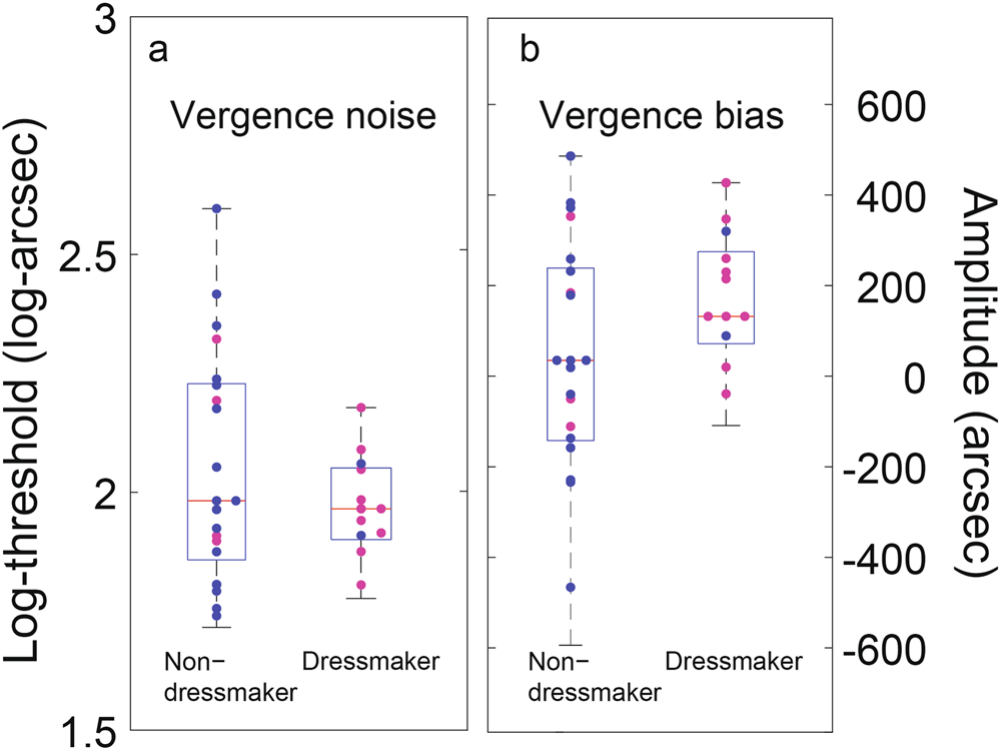
Boxplots of log-transformed vergence thresholds ((**a**), noises) and vergence biases (**b**) from Nonius - line method, for non-dressmaker and dressmaker groups. Medians are in red and the blue box defines the Q1 and Q3 quantiles. The whiskers encompass the entire distribution. Each pink dot is a data point for a female participant and each blue dot is a data point for a male participant.

Neither vergence noise (log-thresholds - Fig. 3; t-test T(32) = 1.13, p = 0.27) nor vergence bias (Fig. 3; t-test T(32) = 1.64; p = 0.11) differed significantly between dressmaker and non-dressmaker groups.

## Discussion

Dressmakers demonstrate a better overall disparity acuity than non-dressmakers for both absolute and relative disparities. There are two plausible and non-exclusive explanations for the dressmakers’ superior stereo-acuity: selection and experience. First, it is possible that having a high stereo-acuity is highly advantageous for becoming a professional dressmaker. We know that observers differ substantially in the precision of their stereo-acuity^49^. Thus, it could be that dressmaking selects for those individuals endowed with superior stereo-acuity because it makes the dressmakers’ task easier, highlighting an example of the functional importance of stereo-vision.

A second plausible explanation is that dressmakers, who spend significant time manually sewing, become accustomed to situations in which they deal with precise visual details and in which the depth matters: sewing requires the dressmaker to put a needle behind or in front of a thread or a cloth. In addition, sewing likely provides immediate and direct feedback when an error is accompanied by negative reinforcement (pain from being pricked by the needle), which may aid perceptual learning. In other words, this could be a form of stereo-plasticity from a manual task. For that reason, it is important to note that our dressmakers were selected because they were hand sewing rather than machine sewing. Interestingly, this interpretation, if confirmed, would also imply that absolute disparity acuity (or readout) can be improved by experience.

With the present cross-sectional design, it is not possible to know whether dressmakers’ acuities are better because of learning by experience, or because of an implicit selection for better stereoscopic vision by the profession. The two possible origins of the effect could also be cumulative. In the future, a training study could be carried out to address this issue. A recent study demonstrates a complementary idea: namely, that studying representative arts, a profession involving mostly 2D images, is associated with poorer stereopsis^50^. That study also cannot disentangle between a training effect (while ignoring stereo 3D-information in order to represent 2D) and a selection bias (when impaired stereo 3D-vision helps to represent 2D information).

Interestingly, we found no difference between groups for the vergence precision and accuracy (vergence noise and bias). This was not a given, as sewing, which requires high precision, would certainly benefit from better vergence, and may also provide a form of vergence training.

Finally, the lack of interaction between the type of disparity (absolute or relative) and the type of observer (dressmaker or non-dressmaker) is consistent with the view that relative disparities are calculated from absolute disparities^41, 46, 47^. In this view, improvements in threshold may have originated at the level of the absolute disparity encoding, and then percolated to relative disparities. Note that improved absolute stereo-acuities could be expected to result in improved vergence, as absolute disparities contribute to vergence noise^42–45^. Yet, similar vergence was measured across groups. This is probably owing to the fact that there are other sources of vergence noise than absolute disparity noise. Among such sources are motor noise and noise in the estimation of the vergence angle from eye muscle tension. Those sources of noise may constitute greater limiting factors for vergence fixation than the absolute disparity noise.

We acknowledge that our two groups differed in gender balance with the dressmaker group being predominantly female and the non-dressmaker group male. To further assess a potential gender difference in our sample, we show on each figure which participant is a female or a male (color-coded). No clear pattern in favour of a gender bias appears, with around half of the women in the non-dressmaker group, and half of the men in the dressmaker group fall on either side of the median line (Figs 2 and 3). If anything, male participants in the dressmaker group had slightly better stereo-acuity. In addition and importantly, several large - scale studies have investigated gender differences in stereo-acuity and they reported no differences, both for standardized clinical tests^51^ and psychophysical measures^52^. Therefore, gender is unlikely to explain the effect we document here.

Although all of our participants successfully passed the Randot and Butterfly clinical stereo-tests with an acuity better than 70 arcsec, we were unable to measure relative-disparity stereo-acuity better than 3000 arcsec for four of them (out of 34) with our psychophysical method, which had a greater range and sensitivity than either clinical test. This suggests that clinical measures may still present monocular cues^4^. While clinical tests can be performed quickly, allowing large-scale screening tests, they are unsuitable for detecting group differences of the size we document here. There is a clear need for new, computerized tests of stereopsis for the clinic, that contain no monocular cues, and we are encouraged that some, such as “Asteroid” are currently being developed (J. Read, personal communication).

To conclude, we were interested in the role of expertise in the perception of stereoscopic depth. We have shown that dressmakers have better stereoscopic acuity than non-dressmakers for both absolute and relative disparities, and no difference in their vergence abilities. The findings are compatible with two non-exclusive possibilities: either that stereopsis has a clear functional importance (here, to success in dressmaking), or that experience with fine manual tasks can influence the precision of the stereoscopic system. Only a training study could disentangle the two options, with one of them opening a door to new ways of training stereoscopic vision.

## Methods

The stimuli, methods and data have been described in detail elsewhere^41, 53^, therefore we simply provide a brief overview below.

### Observers

Thirteen professional dressmakers (11 female, 2 male, age range: 21–34 years, average: 27.6) and twenty-one non-dressmakers (4 female, 17 male, age mean: 24.1, age range: 19–35 years) participated in the study. Only dressmakers with substantial experience (minimum 2 h/week over the last 3 years) with manual sewing (rather than machine sewing) were included in the study. None of the observers had ever participated in visual studies.

Crossed stereoacuity was better than or equal to 70 arcsec on two clinical stereo-tests (Randot circle test and Butterfly circle test) for all observers. All passed the random dot stereogram part of each test. Following recommendations in ref. 54, we report exclusion of participants at the first stage: no participant was excluded at the clinical stereo-test stage. We collected informed consent for all participants and they all obtained monetary compensation for their participation.

Both groups were fully naïve about the computerized tasks in our study. The study was carryout out in accordance with the Declaration of Helsinki and was approved by UNIGE’s Ethics Committee.

### Stereo-task stimuli and procedures

The two stereo tasks that we used to collect thresholds for absolute and relative disparities, used nearly identical stimuli (vertical white lines 20-arcmin long and 26-arcsec wide on a black background). We presented stereoscopic stimuli appearing in depth using a stereoscope in a darkened room. Distance to the screen was 2.1 m and we used a subpixel presentation technique so that binocular disparities as small as 2.6 arcsec could be reliably presented on screen (for full details, see ref. 41).

A trial started when the fixation point was presented. Observers had to fixate it, and to maintain precise vergence. Vergence feedback was achieved through the perceived horizontal alignment of Nonius lines around the fixation. After aligning the nonius lines, the observer pressed a key which initiated the disappearance of all items on the screen and replaced them with a 10-ms mask made of uniform uncorrelated white noise. The two vertical lines of the stereoacuity stimulus were then presented for 200 ms. Vergence eye movements were precluded by the short presentation time. To minimize the effect of monocular cues, a horizontal jitter in the position of both lines was added. A black screen then replaced the stimulus.

### Absolute disparity task

Participants were shown the two vertical lines at the same depth. We measured the *absolute* disparity thresholds using the method of single stimuli with an implicit reference^55–57^. For each trial, observers had to decide whether the depth between the (extinguished) fixation point and the lines was smaller or larger than the mean of the same depth over all previous trials seen in the block. The method allowed us to minimize differences in memory load inherent to the task. We estimated that the largest stereo-threshold that we could reliably measure was 3000 arcsec, using a Monte Carlo experiment simulating an ideal observer (2000 repetitions). Larger thresholds could be measured but were most likely under-estimated.

### Relative disparity task

Participants were shown the two vertical lines identical to those in the absolute disparity task. However, when measuring *relative* disparity thresholds, each line presented a different depth and observers responded about the depth distance *between* the lines. Observers had to decide whether the depth difference between the two lines was smaller or larger than the mean of the same depth over all trials seen in the block.

### Vergence measures

We measured vergence using the Nonius line method described in ref. 41. In short, participants were presented with stimuli as identical as possible as the disparity task stimuli. After the initial fixation Nonius lines, whose goal was to ensure the best vergence fixation, another set of Nonius lines was dichoptically flashed with some horizontal jitter. The lines were also shifted horizontally from each other and the shift was varied with a staircase procedure. The task was to judge whether the line above (extinguished) fixation was flashed to the left or to the right of the line below. Vergence data was separated in two aspects: the noise (which reflects the variability of vergence) and the bias (which reflects the accuracy of vergence).

### Statistical Analyses

All statistical tests were conducted at criterion α = 0.05, with n = 34. In the absolute disparity task, participants ran a block with a reference at a 5-arcmin disparity and the other with the reference at 10 arcmin. The blocks did not differ significantly (mixed ANOVA model with absolute disparity condition as a within-subject factor, and group as another factor: F(1,32) = 0.47; p = 0.50; and on log scale: F(1,32) = 0.63; p = 0.43). Therefore, we merged them for the rest of the analyses.

When studying acuities in absolute and relative disparity tasks, Lilliefors tests showed that the samples were not normally distributed (absolute disparity condition: p = 0.012 for the control group and p = 0.0021 for the dressmaker group; relative disparity condition: p = 0.0015 for control group and p = 0.001 for dressmaker group). However, the log-transformed distributions could not be shown to diverge from normality, using Kolmogorov-Smirnov or Lilliefors tests (all p > 0.22). Cochran test on the log-transformed thresholds demonstrated that the assumption of homoscedasticity was met for all samples (C = 0.31; p = 0.75), therefore we used log-transformed stereo-thresholds.

For the stereo-threshold analysis, we use a modified Thompson tau procedure (median as central value, alpha = 0.01), which identified two observers as outliers (one in the non-dressmaker group/absolute disparity condition and one in the dressmaker group/relative disparity condition). Their values were replaced with the group median in each condition.

Vergence - noise estimates for the non-dressmaker group were not normally distributed (Lilliefors test, p = 0.0011). Log-transformed thresholds were not different from Gaussian distributions (using both Kolmogorov-Smirnov and Lilliefors tests, all p > 0.11), therefore we used log-transformed data for the vergence noise analysis. Outlier detection (modified Thomson tau) also detected 2 outliers (1 in each group). Their values were replaced with the group median in each condition.

Distributions of vergence biases were not different from Gaussian distributions (using both Kolmogorov-Smirnov and Lilliefors tests, all p > 0.50) and therefore we used raw data for the analysis. Outlier detection (modified Thomson tau) also detected 4 outliers (2 in each group). Their values were replaced with the group median in each condition.

### Data Availability

The dataset is available online on Figshare public repository.
